# Need to optimise infant feeding counselling: A cross-sectional survey among HIV-positive mothers in Eastern Uganda

**DOI:** 10.1186/1471-2431-9-2

**Published:** 2009-01-09

**Authors:** Lars T Fadnes, Ingunn MS Engebretsen, Henry Wamani, Jonathan Wangisi, James K Tumwine, Thorkild Tylleskär

**Affiliations:** 1 Centre for International Health, University of Bergen, Bergen, Norway; 2 Department of Paediatrics and Child Health, Makerere University, Kampala, Uganda; 3 School of Public Health, Makerere University, Kampala, Uganda; 4 TASO-Mbale, Mbale, Uganda

## Abstract

**Background:**

The choice of infant feeding method is important for HIV-positive mothers in order to optimise the chance of survival of their infants and to minimise the risk of HIV transmission. The aim of this study was to investigate feeding practices, including breastfeeding, in the context of PMTCT for infants and children under two years of age born to HIV-positive mothers in Uganda.

**Methods:**

In collaboration with The Aids Support Organisation Mbale, we conducted a cross-sectional survey involving 235 HIV-positive mothers in Uganda. Infant feeding practices, reasons for stopping breastfeeding, and breast health problems were studied. Breastfeeding duration was analysed using the Kaplan-Meier method based on retrospective recall.

**Results:**

Breastfeeding was initiated by most of the mothers, but 20 of them (8.5%) opted exclusively for replacement feeding. Pre-lacteal feeding was given to 150 (64%) infants and 65 (28%) practised exclusive breastfeeding during the first three days. One-fifth of the infants less than 6 months old were exclusively breastfed, the majority being complementary fed including breast milk. The median duration of breastfeeding was 12 months (95% confidence interval [CI] 11.5 to 12.5). Adjusted Cox regression analysis indicated that a mother's education, socio-economic status, participation in the PMTCT-program and her positive attitude to breastfeeding exclusively, were all associated with a reduction in breastfeeding duration. Median duration was 3 months (95% CI 0–10.2) among the most educated mothers, and 18 months (95% CI 15.0–21.0) among uneducated mothers. Participation in the PMTCT program and being socio-economically better-off were also associated with earlier cessation of breastfeeding (9 months [95% CI 7.2–10.8] vs. 14 months [95% CI 10.8–17.2] and 8 months [95% CI 5.9–10.1] vs. 17 months [95% CI 15.2–18.8], respectively). The main reasons for stopping breastfeeding were reported as: advice from health workers, maternal illness, and the HIV-positive status of the mother.

**Conclusion:**

Exclusive breastfeeding was uncommon. Exclusive replacement feeding was practised by few HIV-positive mothers. Well-educated mothers, mothers who were socio-economically better-off and PMTCT-attendees had the shortest durations of breastfeeding. Further efforts are needed to optimise infant feeding counselling and to increase the feasibility of the recommendations.

## Background

Widespread promotion of exclusive breastfeeding could prevent child mortality by 8% [1]. However, transmission of HIV through breast milk has made breastfeeding counselling more complicated in low-income countries where HIV is prevalent.

Every year, more than half a million infants become infected with HIV. These infection rates are disproportionately distributed geographically; mother-to-child-transmission, in the context of antiretroviral prophylaxis is below 1% in Europe and the USA, but exceeds 30% in many poorly resourced countries, with Sub-Saharan Africa carrying the highest burden [2,3]. The incidence of HIV infection among children has fallen in many areas, but this seems to be more related to a reduction of the HIV prevalence among mothers than gains in the PMTCT program [4].

In many countries where HIV is prevalent, the infant mortality rate is high. Considering the risks of both infant mortality and HIV transmission, breastfeeding is strongly correlated with a higher HIV-free child survival rate compared to formula feeding where the infant mortality rate is above 4% [5,6]. Exclusive breastfeeding can be associated with higher HIV-free survival at 6 months than mixed feeding [6,7]. Post-natal vertical HIV transmissions increase with a longer breastfeeding duration [8]. Cessation of breastfeeding at the age of 6 months has consequently been recommended wherever replacement feeding at that age is acceptable, feasible, affordable, sustainable and safe [8,9]. Failure to sustain replacement feeding and re-introduction of breastfeeding after initial cessation is particularly risky in terms of mother-to-child transmission of HIV [10]. Infant feeding recommendations for HIV-positive mothers are confusing and have resulted in disadvantageous feeding patterns and mixed feeding in particular [11]. Mixed feeding is associated with a higher morbidity and mortality risk than exclusive breastfeeding for infants of both HIV-positive and HIV-negative mothers, and with increased HIV transmission from HIV-positive mothers [6,7,12-16]. Exclusive breastfeeding is also associated with a reduced risk of breast health problems [17].

The frequencies with which exclusive breastfeeding, mixed feeding, prelacteal feeding and replacement feeding are practised differ widely throughout Africa [18]. Region-specific measurements are essential to develop regional-specific recommendations.

We have investigated infant feeding practices, including breastfeeding, for infants and children under 2 years of age born to HIV-positive mothers in Mbale, Eastern Uganda.

## Methods

### Study area

The study was conducted during 2005 in Eastern Uganda in collaboration with The Aids Support Organisation (TASO). TASO is the largest national-based non-governmental organisation working with HIV-positive people in Africa. It is a grassroots movement providing counselling, information, support and medical treatment.

The study area included Mbale district, together with areas accessed through TASO Outreach Clinics in adjacent regions in the districts of Sironko, Pallisa and Kumi. Mbale district has a population of 720,000 of predominantly Bagisu people, with 90% living as subsistence farmers in rural environments. The overall literacy rate is 64% for men and 49% for women [19]. Uganda has an HIV prevalence of 7.5% in women aged 15–49 years (2005) [20]. In the period of the study, introduction of routine HIV counselling and testing was starting in Ugandan hospitals [21]. The acceptance of testing had increased substantially. The national PMTCT program was introduced in Uganda first as a pilot in 1998 and more widely in 2001 [22].

This cross-sectional study collected information from 240 HIV-positive mothers with children aged 0–23 months. All mothers were recruited from TASO by consecutive sampling, and they participated voluntarily with informed consent. No mother contacted as a potential interviewee refused to participate. Five mother-infant pairs were excluded from the study because of missing information or the child was over 23 months old. Accordingly, 235 HIV-positive mothers were included in the study. Three pairs of data collectors who were fluent in Lumasaba (the local language), Luganda (the language of the central region) and English conducted the interviews with the mothers.

To check the reliability, 15 mothers were re-interviewed by another pair of data collectors 2–4 weeks after the initial interview. The answers showed only minor discrepancies and a high degree of consistency.

### Questionnaire

The structured interview contained topics concerning breastfeeding and feeding habits, feeding knowledge, mother's and father's education, occupation, household assets, time of HIV diagnosis, self-rated health [23], mother-rated health of infant and PMTCT program participation. A list of 30 liquid, semi-solid and solid foods was utilized with 24-hour recall, 1-week recall and recall since birth. Using a symptom-based semi-quantitative approach, we examined breastfeeding problems and reasons for stopping breastfeeding. The questionnaire was pre-tested to ensure correct understanding of the questions.

### Data handling

Double entry was done in EpiData 3.1, and SPSS 14 was used for data analysis.

### Definitions

Feeding information was based on World Health Organisation (WHO) definitions and recommendations [24], as follows. Exclusive breastfeeding: giving breast milk only, except for medicines and vitamin or mineral supplements; predominant breastfeeding: breast milk is nutritionally dominant, but with the possible addition of water-based fluids, fruit juices, tea without milk or oral rehydration salts; complementary feeding including breast milk (often referred to as mixed feeding): non-human milk, semi-solids or other solids given in addition to breast milk; replacement feeding: breastfeeding stopped or never being given any breast milk. Exclusive replacement feeding was defined as never having given any breast milk. Pre-lacteal feeding was defined as any food item or liquid other than breast milk given to the infant during the first 3 days after delivery.

### Ethics

Ethical approval was obtained from Makerere University, Faculty of Medicine Ethics and Research Committee, and the Uganda National Council for Science and Technology. Informed consent was obtained from each participant.

### Statistics

Baseline characteristics were examined with frequency tables. Feeding patterns were compared using χ^2 ^statistics. Consistency was checked by Cohen's Kappa statistic.

The participants were grouped socio-economically into quintiles based on wealth assessment using principal component factor analysis [25]. Housing characteristics and assets including toilet facilities, number of rooms and beds, roof material, lantern, radio, television, bicycle and vehicles were included in the model. The quintiles were based on the first principal component, a recognised method as a good proxy for household wealth [26]. Breastfeeding duration in this cross-sectional study was estimated using Kaplan-Meier survival analysis with a Mantel-Cox log rank test to compare the estimates. Self-reported breastfeeding duration is used in the model. Cox regression analysis was used to estimate the independent impact of each factor on breastfeeding duration. Co-linearity, hazard plots and residual plots were checked. A backward "conditional" regression model was used in the multivariate analysis with removal set at a 0.1 level of significance.

## Results

The median maternal age was 30 years (inter-quartile range [IQR] 28–35) (Table 1). The age distribution of the infants and children was: 0–5 months: 37 infants; 6–11 months: 53 infants; 12–17 months: 65 children; 18–23 months: 80 children [see Additional file 1]. Median maternal education was 5 years of schooling (IQR 3–7). Fathers were more educated than mothers with a median of 7 years of education (IQR 5–10). Half the mothers were widowed.

**Table 1:** Median breastfeeding duration with Kaplan-Meier analysis including all the infants (n = 235) and a Mantel-Cox log rank test to compare ranking of the estimates

	n (%)	Median in months	95% CI	Log Rank test (Mantel-Cox)
**Mother's education**				*χ^2 ^= 24.0 (4 df^a^)*
None	27 (11)	18	15.0 – 21.0	*p *< 0.001
Stopped in primary	125 (53)	14	12.1 – 15.9	
Completed primary (7 years)	39 (17)	12	8.0 – 16.0	
Secondary education	37 (16)	8	6.8 – 9.2	
Higher education (≥ 12 years)	7 (3)	3	0 – 10.2	
				
**Father's education**				*χ^2 ^= 11.4 (4 df)*
None	14 (6)	^b^	^b^	*p *< 0.05
Stopped in primary	72 (32)	13	11.6 – 14.4	
Completed primary (7 years)	62 (27)	15	11.8 – 18.2	
Secondary education	50 (22)	9	7.3 – 10.7	
Higher education (≥ 12 years)	30 (13)	8	3.8 – 12.2	
				
**Mother's age**				*χ^2 ^= 1.7 (1 df ^ac^)*
≤ 24	22 (10)	18	16.3 – 19.7	*p *= 0.20
25 – 29	61 (26)	12	10.9 – 13.1	
30 – 34	85 (36)	12	11.3 – 12.7	
≥ 35	67 (29)	12	11.3 – 12.7	
				
**Marital status**				*χ^2 ^= 2.5 (2 df)*
Married/cohabiting	91 (39)	12	9.4 – 14.6	*p *= 0.29
Widowed	112 (48)	12	11.4 – 12.6	
Divorced/separated or single	32 (14)	13	4.5 – 21.5	
				
**Socio-economic status**				*χ^2 ^= 9.3 (1 df^c^)*
Bottom quintile, poorest	47 (20)	17	15.2 – 18.8	*p *< 0.01
2nd quintile	47 (20)	12	11.3 – 12.7	
3rd quintile	46 (20)	9	6.0 – 12.0	
4th quintile	48 (20)	16	11.4 – 20.6	
Top quintile, least poor	46 (20)	8	5.9 – 10.1	
				
**Mother's work**				*χ^2 ^= 7.5 (1 df)*
Farming	201 (86)	12	10.9 – 13.1	*p *< 0.01
Do not farm	34 (14)	8	6.9 – 9.1	
				
**Living area**				*χ^2 ^= 3.3 (1 df)*
Rural	205 (87)	12	11.0 – 13.0	*p *= 0.07
Urban	30 (13)	8	4.7 – 11.3	
				
**HIV-diagnosis**				*χ^2 ^= 10.1 (1 df)*
After delivery	85 (36)	15	10.5 – 19.5	*p *< 0.01
Before delivery	150 (64)	12	10.4 – 13.6	
				
**Participation in the PMTCT-program**				*χ^2 ^= 17.5 (1 df)*
Did not attend	107 (46)	14	10.8 – 17.2	*p *< 0.001
Attended	128 (54)	9	7.2 – 10.8	
				
**Anyone talked about breastfeeding**				*χ^2 ^= 12.6 (1 df)*
No	44 (19)	18	15.7 – 20.3	*p *< 0.001
Yes	191 (81)	12	11.5 – 12.5	
				
**Mothers' self-rated health**				*χ^2 ^= 0.1 (1 df^c^)*
Very healthy	33 (14)	12	9.5 – 14.5	*p *= 0.73
Quite healthy	138 (59)	12	11.4 – 12.6	
Not very healthy	64 (27)	14	9.7 – 18.3	
				
**Mothers'-rated health of child**				*χ^2 ^= 0.4 (1 df^c^)*
Very healthy	37 (16)	9	5.1 – 12.9	*p *= 0.54
Quite healthy	111 (48)	12	10.1 – 13.9	
Not very healthy	83 (36)	12	10.8 – 13.2	
				
**Belief about 6 months of exclusive breastfeeding**				*χ^2 ^= 9.2 (1 df^c^)*
Sure it would be good	29 (12)	7	5.6 – 8.4	*p *< 0.01
Think it would be good	42 (18)	12	8.6 – 15.4	
Think it would hurt	104 (44)	12	11.0 – 13.0	
Sure it would hurt	59 (25)	15	12.0 – 18.0	

^a^df (degrees or freedom)

^b ^could not be measured (only 3 stopped among 14 cases and 151 months of follow-up)

^c ^linear trend assumed

### Feeding practices

Of 235 HIV-positive mothers, 215 (91.5%) initiated breastfeeding while 20 (8.5%) never breastfed their infants. Among 128 mothers who attended the PMTCT program, 18 (14%) avoided breastfeeding completely, while 2 of those not participating did not breastfeed (p < 0.001). Among the attendees and non-attendees in the PMTCT program, the proportions opting for exclusive breastfeeding during the first three days were not significantly different, 28.1% versus 27.1%, respectively. Ten among the 46 mothers in the better-off quintile did not initiate breastfeeding within the first three days. Nine of these 10 (90%) mothers continued with exclusive replacement feeding. Among the poorer 189 mothers, 11 (42%) out of 26 mothers who did not initiate breastfeeding within the first three days continued with exclusive replacement feeding, whereas 15 mothers introduced breastfeeding later on (p < 0.05).

Within the first two hours after delivery, 131 (56%) had initiated breastfeeding, with 178 (76%) having done so within the first day. Pre-lacteal feeding was given by 150 (64%) while 65 (28%) practised exclusive breastfeeding during the first three days. Replacement feeding was practised by only one of the 85 mothers diagnosed with HIV after delivery.

One-week recall and 24-hour recall gave similar results for the infant feeding patterns, giving a Spearman correlation coefficient of 0.96 among infants below 6 months of age and 1.0 above 6 months of age (Table 2). Among the infants less than 6 months old, one-fifth were exclusively breastfed and most were fed complementary including breast milk. Two-thirds of the infants older than 6 months were fed complementary including breast milk and the remaining third were replacement fed. The rate of replacement feeding increased with age. Among children aged 12–17 months, 37 (58%) were replacement fed, while 75 (93%) of children aged 18–23 months were replacement fed.

**Table 2:** Recall comparison of different feeding patterns based on 24-hour, 1-week and since birth recall. N (%) of infants in age range feeding in particular pattern based on specific recall period.

	24-hour recall	1-week recall	Since birth recall
**0 – 5 months**			
Exclusive breastfeeding	9 (24)	8 (22)	5 (14)
Predominant breastfeeding	3 (8)	3 (8)	5 (14)
Complementary feeding incl. breast milk	19 (51)	20 (54)	21 (57)
Replacement feeding	6 (16)	6 (16)	6 (16)
			
**6 – 11 months**			
Exclusive breastfeeding	0 (0)	0 (0)	0 (0)
Predominant breastfeeding	0 (0)	0 (0)	0 (0)
Complementary feeding incl. breast milk	36 (68)	36 (68)	36 (68)
Replacement feeding	17 (32)	17 (32)	17 (32)

0 – 5 months: n = 37; 6 – 11 months: n = 53.

### Breastfeeding duration

**Figure 1:**
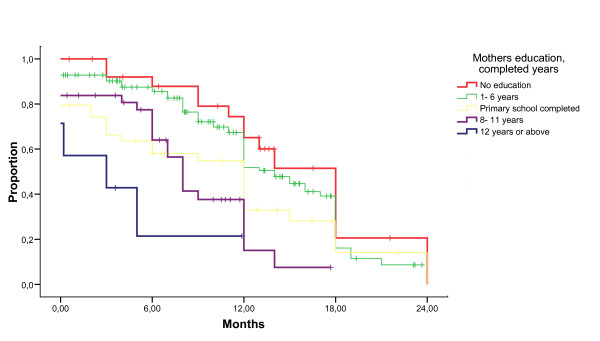
**Breastfeeding duration in months (x-axis) for different groups of mothers based on their education**. Proportion still breastfeeding (y-axis) visualised with a Kaplan-Meier-plot.

**Figure 2:**
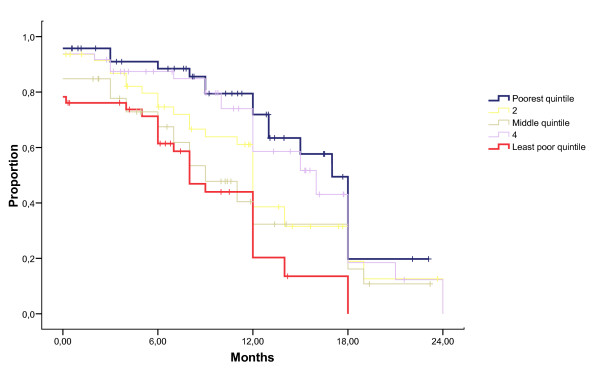
**Breastfeeding duration in months (x-axis) for different socio-economic groups**. Proportion still breastfeeding (y-axis) visualised with a Kaplan-Meier-plot.

The median duration of breastfeeding was 12 months (95% confidence interval 11.5–12.5). Education was associated with a marked reduction in duration of breastfeeding, with a median duration of 3 months (95% CI 0–10.2) among mothers with more than 12 years of schooling, and a median of 18 months (95% CI 15.0–21.0) among mothers lacking education (Figure 1). This effect was seen both with and without adjusting for other factors with Cox regression with a 4.5 and 6.4-fold increase in hazard ratio for breastfeeding cessation, respectively (Table 3). The level of the father's education, whether the mother was a farmer, and the timing of HIV-diagnosis in relation to birth had similar effects on the crude analysis, but not in the adjusted Cox regression analysis. Breastfeeding duration differed substantially among mothers in the poorest and least poor quintiles. The median duration was 8 months (95% CI 5.9–10.1) among the least poor and 17 months (95% CI 15.2–18.8) among the poorest (Figure 2). Mother's age, marital status, rural or urban life, self-rated health of the mother and mother-rated health of the infant were not significantly associated with breastfeeding duration. Mothers counselled in the PMTCT program stopped breastfeeding earlier than those who did not attend the program. Duration of breastfeeding was shorter among mothers who had discussed breastfeeding with someone compared to those who had not. Those who considered exclusive breastfeeding to be beneficial for the infant, stopped breastfeeding earlier compared to those who considered it harmful. In a restricted analysis including children above 18 months of age, the median duration of breastfeeding was 12 months (95% CI 11.4–12.6).

**Table 3:** Cox regression of breastfeeding cessation, unadjusted and adjusted hazard ratio (HR). Only factors in the final adjusted model have HR estimates (right-hand columns).

	Breastfeeding cessation, unadjusted (crude)		Breastfeeding cessation, adjusted	
	HR	95% CI	HR	95% CI
**Mother's education**				
None	1		1	
Stopped in primary	1.3	0.7 – 2.4	1.3	0.7 – 2.5
Completed primary (7 years)	1.9*	1.0 – 3.7	2.1*	1.0 – 4.2
Secondary education	3.0*	1.5 – 6.0	2.4*	1.1 – 5.0
Higher education (≥ 12 years)	6.4*	2.2 – 18	4.5*	1.4 – 15
				
**Father's education**				
None	1			
Stopped in primary	1.7	0.6 – 4.8		
Completed primary (7 years)	1.7	0.6 – 4.9		
Secondary education	3.0*	1.1 – 8.4		
Higher education (≥ 12 years)	3.0	1.0 – 8.8		
				
**Mother's age**				
≤ 24	1			
25 – 29	1.8	0.8 – 3.7		
30 – 34	1.7	0.9 – 3.5		
≥ 35	1.8	0.9 – 3.7		
				
**Marital status**				
Married/cohabiting	1			
Widowed	1.2	0.9 – 1.8		
Divorced/separated or single	1.5	0.8 – 2.6		
				
***Socio-economic status***				
Bottom quintile, poorest	1		1	
2nd quintile	1.8	1.0 – 3.2	2.4*	1.2 – 4.8
3rd quintile	2.2*	1.2 – 4.0	2.6*	1.3 – 5.1
4th quintile	1.3	0.7 – 2.4	1.1	0.5 – 2.3
Top quintile, least poor	3.1*	1.7 – 5.5	3.0*	1.5 – 6.0
				
**Mother's work**				
Farming	1			
Do not farm	1.8*	1.1 – 2.7		
				
**Living area**				
Rural	1			
Urban	1.5	0.9 – 2.5		
				
**HIV-diagnosis**				
After delivery	1			
Before delivery	1.7*	1.2 – 2.4		
				
**Participation in the PMTCT-program**				
Did not attend	1		1	
Attended	2.0*	1.4 – 2.8	2.0*	1.3 – 3.0
				
**Anyone talked about breastfeeding**				
No	1		1	
Yes	2.2*	1.4 – 3.7	1.9*	1.1 – 3.3
				
**Mothers self-rated health**				
Very healthy	1.1	0.6 – 1.9		
Quite healthy	1.1	0.8 – 1.7		
Not very healthy	1			
				
**Mothers-rated health of child**				
Very healthy	1.3	0.7 – 2.1		
Quite healthy	1.0	0.7 – 1.4		
Not very healthy	1			
				
**Belief about 6 months of exclusive breastfeeding**				
Sure it would be good	2.8*	1.5 – 5.0	3.1*	1.6 – 5.8
Think it would be good	1.3	0.7 – 2.2	1.7	1.0 – 3.1
Think it would hurt	1.3	0.9 – 2.1	2.0*	1.2 – 3.2
Sure it would hurt	1		1	

*p < 0.05 (group is significantly different from reference group)

HR (hazard ratio)

### Reasons for cessation of breastfeeding

Breastfeeding had been stopped by 116 mothers at the time of the interview. Advice from health workers, illness of the mother, and the fact that the mother was HIV-positive were reported as the main reasons for stopping breastfeeding (Table 4). Other reasons for stopping breastfeeding were: breastfeeding difficulties, perceived insufficient milk production, the notion that the child was "old enough" or "big enough", and that the child could eat without help. Family pressure, work and new pregnancies were reported by only a few mothers. Those stopping breastfeeding before the infant was six months old gave similar reasons to all the mothers stopping breastfeeding.

**Table 4:** Reasons for stopping breastfeeding

	Main reason	Additional reasons ^a^	Total ^a^
	n (%)	n (%)	n (%)
Health workers advice	32 (28)	22 (19)	54 (47)
Illness (weakness, body pain etc)	29 (25)	23 (20)	52 (45)
HIV-diagnosis	20 (17)	54 (47)	74 (64)
Not enough milk	10 (9)	16 (14)	26 (22)
Breastfeeding difficulties	10 (9)	5 (4)	15 (13)
Custom related (e.g. grown "big enough")	9 (8)	11 (9)	20 (17)
Family pressure	2 (2)	0 (0)	2 (2)
New pregnancy	2 (2)	0 (0)	2 (2)
Work situation	1 (1)	4 (3)	5 (4)
Other reasons	1 (1)	4 (3)	5 (4)

Total	116 (100)		

^a ^Reasons other than main reason reported to be important for the choice to stop breastfeeding. 16 (14%) reported main reason only, 66 (57%) reported one additional reason, 28 (24%) reported two additional reasons and 6 (5%) reported three additional reasons.

Fewer than half the breastfeeding mothers experienced problems relating to breastfeeding [see Additional file 2]. A fifth had problems with breastfeeding related to illnesses, such as generalised pain, frequent fever and a feeling of weakness. Breast pain, sore and cracked nipples, and swelling of the breast also burdened 40 (19%) mothers. Three mothers (1%) were diagnosed with mastitis or breast abscess.

## Discussion

This study shows that exclusive breastfeeding was uncommon among the HIV-positive women who opted to breastfeed. Thus, most infants received complementary feeding including breast milk from a very early age, which is an unfavourable situation. Our findings in Uganda agree with studies in other parts of Africa among both HIV-positive mothers and the general population [18,27]. A positive finding is that breastfeeding duration is shortened by many HIV-positive mothers, especially among the well educated, the socio-economically better-off, and those who have attended the PMTCT program or discussed infant feeding with someone. Well-educated mothers breastfed for ~1 year less than their uneducated peers. Whether the shortened breastfeeding duration had any negative effects on the children was not assessed in our study. A recently published randomised study from Zambia indicates that early abrupt weaning of breastfeeding does not significantly reduce HIV-free mortality rates [28]. In addition, prolonged breastfeeding gave a higher survival rate for HIV-positive children compared to those weaned early. HIV screening using a dried blood spot from infants was a feasible approach to the early identification of HIV-positive infants who may benefit from prolonged breastfeeding [29].

Exclusive breastfeeding of infants under 6 months old was less commonly practised among HIV-positive mothers than among the general population reported in the DHS-study in Uganda – 24% of HIV-positive mothers and 63.2% of the general population mothers according to the 24-hour recall data [18]. Similarly, a study of the general population in the same area also reported higher rates of exclusive breastfeeding [30]. Is this an effect of information about the risk of HIV transmission through breastfeeding reaching the HIV-positive mothers? Counselling on infant feeding in many African countries, including Uganda, has been reported to be suboptimal and may be one of the important reasons for the widespread practice of complementary feeding including breast milk [31]. Health workers often overestimated the risk of HIV transmission through breastfeeding and many gave the impression that HIV transmission from mother-to-child is nearly universal [22,31]. Based on key informant interviews, our impression was that replacement feeding was promoted more strongly in the national PMTCT program, whereas exclusive breastfeeding seemed to be more counselled in the non-governmental organisations working with HIV (unpublished data). Although breastfeeding duration was shorter among participants in the national PMTCT program than those not participating, we did not see differences in the rates of exclusive breastfeeding. Another explanation for the dominance of complementary feeding including breast milk may be the fear of making the infant totally reliant on breast milk, which could be particularly true for HIV-positive mothers [32].

In rural and semi-urban HIV-positive mothers in Eastern Uganda, replacement feeding was uncommon. This agrees with a Tanzanian study where replacement feeding with infant formula or cow's milk was seen as unacceptable or infeasible [11]. Introduction of breastfeeding after initial replacement feeding was common except among the socio-economically better-off. Initiating breastfeeding after abrupt weaning is associated with increased viral loads of breast milk, and consequently could be hazardous [10].

Only 56% of the mothers initiated breastfeeding within the first two hours after delivery and 76% initiated it within the first day. A study in Ghana indicated a 2.4-fold increase in risk of neonatal death among infants for whom breastfeeding was not begun within the first day compared to those for whom it was [15]. The authors of that study calculated that 16.3% of neonatal deaths could have been prevented if all neonates had been breastfed within the first hour. Pre-lacteal feeding was given by 64% of the HIV-positive mothers in our study. Not breastfeeding exclusively during the first days has also been shown to increase neonatal mortality [15,33].

Although being HIV-positive was a major reason for stopping breastfeeding by 64% of the mothers, the median breastfeeding duration was 12 months. Breastfeeding duration among HIV-positive mothers was clearly shorter than among the general population, which in the DHS-study in Uganda was 19.9 months [18]. It may seem counterintuitive that mothers perceiving exclusive breastfeeding to be beneficial were breastfeeding for a shorter time than mothers considering exclusive breastfeeding to be harmful. We interpret this as an indication that counselling had some impact, both in terms of increasing knowledge of infant feeding and of influencing behaviour.

Self-reported breastfeeding problems were similar to those given by mothers in the general population in the same area of Uganda, and were slightly more common than among HIV-positive and general population mothers in South Africa [17,34]. The low rate of exclusive breastfeeding probably contributed to the higher proportion of breastfeeding problems. Mothers with breast health problems have a greatly increased risk of infecting their children with HIV [17,35]. Data from South Africa indicate a greater than threefold risk of transmitting HIV from mother to infant when the mother had a serious breast health problem. Similarly, any breast health problems show an increased hazard ratio for HIV transmission compared to the absence of such problems [17].

The cross-sectional design of the study inherently left out diseased children. A similar cross-sectional study in 2003 provided a comparative group in the general population [30]. A limitation of this study is that Kaplan-Meier and Cox regression analyses were conducted in a population where half the infants were censored at the last time-point due to ongoing breastfeeding, which may limit the precision of our estimates and could introduce bias. However, we observed similar associations in an analysis restricted to children with at least 18 month follow-up, and with much lower censoring rates. A third of the HIV-positive mothers acquired their HIV-status after delivery, which might influence the Cox regression analysis compared to the situation where all mothers were diagnosed HIV-positive before delivery. A restricted analysis excluding the mothers acquiring their HIV-diagnosis after delivery gave similar results in the Cox regression compared to when all mothers were included (not published). The use of anti-retroviral medicines was not recorded, but was not common even if roll-out started approximately at the time as the study in Mbale. The recall setting in this study was not optimal, and there may also have been socially desirable answers. It has been suggested that dietary recall once a week has high sensitivity and specificity for exclusive breastfeeding and other feeding patterns to a given age [36]. In our study, recall periods of 24 hours and 1 week yielded similar results. Some studies have indicated that breastfeeding duration is overestimated to an escalating degree with increasing age [37,38], while others have stated that breastfeeding duration is accurately reported [39]. The fact that there was full agreement about breastfeeding duration between the initial and re-interviews reduces the likelihood that this measurement was significantly biased. The fact that all mothers were recruited through TASO may have caused a socio-economically skewed selection. We still feel confident that the data are representative of a large proportion of HIV-positive women in the region in Uganda.

There was a wide difference between the infant feeding practices in this group and WHO recommendations. Infant feeding recommendations for HIV-positive mothers have been confusing [11], which might explain the shortcomings of the practices. More beneficial practices among the well educated is a reason to increase the level of education, while also putting more efforts into counselling of less well-educated mothers.

## Conclusion

Well-educated mothers breastfed for a substantially shorter time than their less well-educated peers. Mothers who were socio-economically better-off or had participated in the PMTCT program also breastfed for shorter durations.

Except among a limited group in this population, replacement feeding was not considered a realistic option in this rural setting. Complementary feeding including breast milk was the dominant practice for infants under 6 months old among the HIV-positive mothers.

There still seems to be many obstacles to optimal infant feeding. Further efforts are needed to optimise counselling on infant feeding and increase the implementation of the recommendations.

## Abbreviations

CI: confidence interval; DHS: Demographic and Health Surveys; HIV: human immunodeficiency virus; IQR: inter-quartile range; PMTCT: Prevention of Mother-to-Child Transmission; SPSS: Statistical Package for the Social Sciences; TASO: The Aids Support Organisation; WHO: World Health Organisation.

## Competing interests

The authors declare that they have no competing interests.

## Authors' contributions

LTF: design, implementation, analysis and writing. IMSE: design, analysis and co-writing. HW: analysis and co-writing. JW: implementation of the study and co-writing. JKT: initiation of the study and co-writing. TT: initiation of the study, design, implementation, analysis and co-writing.

## Pre-publication history

The pre-publication history for this paper can be accessed here:



## Supplementary Material

Additional file 1 **Infant age histogram; age in months**. Age distribution of infants at the time of the interview represented with histogram. Click here for file

Additional file 2 **Reported breastfeeding problems among 215 breastfeeding mothers.** Frequency of breastfeeding problems among 215 breastfeeding mothers. Click here for file
