# Supplementary figures and images for: Need to optimise infant feeding counselling: A cross-sectional survey among HIV-positive mothers in Eastern Uganda
Source: BMC Pediatr. 2009 Jan 9;9:2. doi: 10.1186/1471-2431-9-2 (PMC2657132; doi:10.1186/1471-2431-9-2)

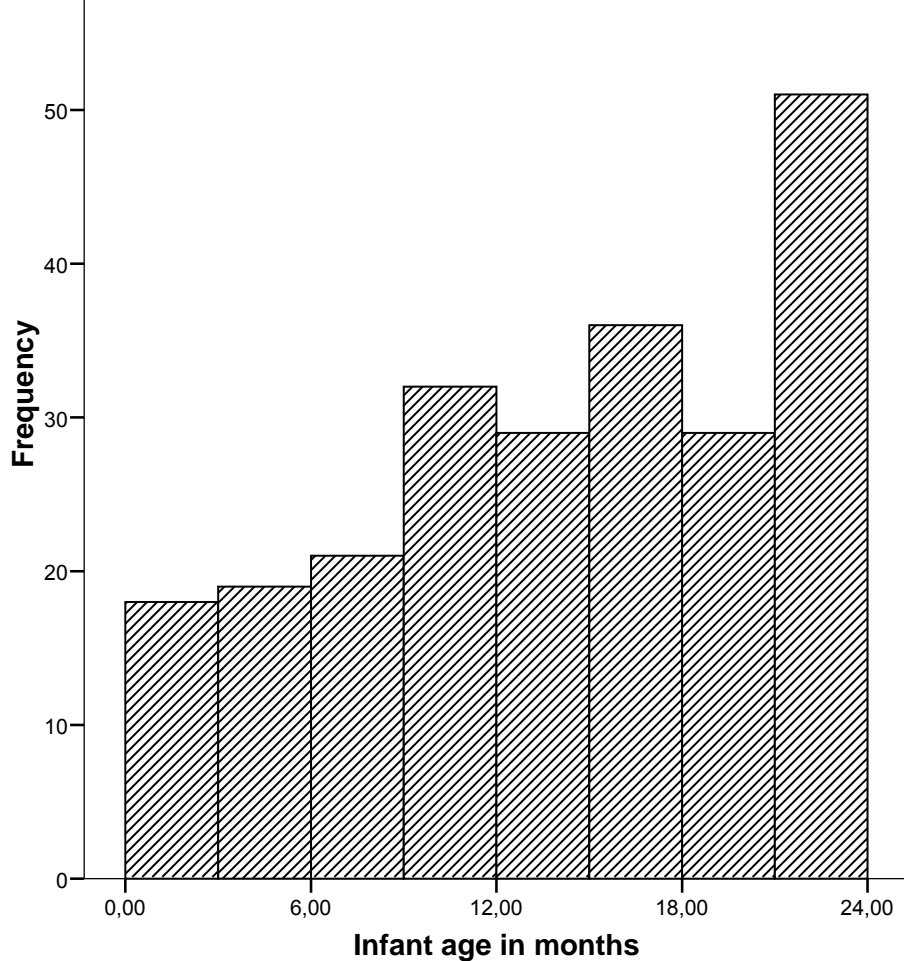

Mean =13,5021 □  
Std. Dev. =6,89133 □  
N =235

Supplement: Additional file 1 — Infant age histogram; age in months. Age distribution of infants at the time of the interview represented with histogram. [file 1471-2431-9-2-S1.pdf]
