# Supplementary material for: Need to optimise infant feeding counselling: A cross-sectional survey among HIV-positive mothers in Eastern Uganda
Source: BMC Pediatr. 2009 Jan 9;9:2. doi: 10.1186/1471-2431-9-2 (PMC2657132; doi:10.1186/1471-2431-9-2)
Supplement: Additional file 2 — Reported breastfeeding problems among 215 breastfeeding mothers. Frequency of breastfeeding problems among 215 breastfeeding mothers. [file 1471-2431-9-2-S2.pdf]

Table 5: Reported breastfeeding problems among 215 breastfeeding mothers

	Main problem n (%)
No problems reported	*112 (52)*
Related to HIV-illness (weakness, body pain etc)	43 (20)
Breast and nipple pain	19 (9)
Sore/ cracked nipple	11 (5)
Volume related/ not enough milk	10 (5)
Swelling of the breast (or nipple)	10 (5)
Mastitis or abscess diagnosed	3 (1)
Other (child related, biting, etc)	7 (3)
Total	215 (100)
